# Specificity of endothelial cells in endothelial dysfunction of diabetic kidney disease and their crosstalk with neighboring cells: an updated review

**DOI:** 10.3389/fendo.2025.1742296

**Published:** 2025-12-17

**Authors:** Jiarui Li, Yue Zhang, Xiao Sun, Huifang Guan, Jingshuo Wang, Shuang Zhao, Chuanxi Tian, Mingda Han, Kaile Ma, Min Li

**Affiliations:** 1 College of Traditional Chinese Medicine, Changchun University of Chinese Medicine, Changchun, China; 2 Guang’ anmen Hospital of China Academy of Chinese Medical Sciences, Beijing, China; 3 The Affiliated Hospital to Changchun University of Chinese Medicine, Changchun University of Chinese Medicine, Changchun, China; 4 Research Laboratory of Molecular Biology, Guang’anmen Hospital of China Academy of Chinese Medical Sciences, Beijing, China

**Keywords:** cell communication, DKD, endothelial dysfunction, glomerular endothelial cells, mesangial cells, podocytes, tubular epithelial cells

## Abstract

Diabetic kidney disease (DKD), a major microvascular complication of diabetes, is characterized by persistent proteinuria, a declining glomerular filtration rate (GFR), and distinctive pathological alterations, often progressing to end-stage renal disease (ESRD). Renal endothelial cells homeostasis is essential for maintaining glomerular architecture and function, sustaining an anti-inflammatory and antithrombotic environment, and preventing fibrosis. Although injury to intrinsic renal cells has been widely studied, the distinct contribution of glomerular endothelial cells (GECs) to endothelial dysfunction and their crosstalk with neighboring cells remain poorly defined. Crosstalk between renal cells is critical for integrity of the glomerular filtration barrier (GFB) and overall renal homeostasis. This review provides an updated and systematic overview of the distinct roles of GECs in DKD, encompassing their phenotypic heterogeneity, early glycocalyx damage with increased vascular permeability, and late-stage renal fibrosis. It further elaborates on the dynamic interactions between GECs and podocytes, mesangial cells (MCs), and tubular epithelial cells (TECs). Additionally, we summarize therapeutic strategies targeting endothelial function in DKD, including direct anti-hyperglycemic interventions, non-glycemic interventions, and approaches focused on endothelial injury biomarkers. In summary, this review highlights the distinct and underappreciated role of endothelial cells in DKD, emphasizing their therapeutic potential as a primary target.

## 1 Introduction

Diabetic kidney disease (DKD) is one of the microvascular complications of diabetes. It is characterized by persistent proteinuria, decreased glomerular filtration rate (GFR), and characteristic pathological changes (such as glomerular basement membrane thickening and mesangial expansion) (1), and often culminates in end-stage renal disease (ESRD) (2). Endothelial surface layer (ESL) degradation is one of the earliest markers of diabetic microvascular injury (3). Hyperglycemia-induced endothelial dysfunction represents an early and initiating event in diabetic vasculopathy (4). During the progression of DKD, cellular injury within the glomerulus can lead to pathological alterations in renal structure and vasculature, ultimately manifesting as abnormal renal hemodynamics such as increased glomerular filtration, renal dysfunction, and elevated proteinuria (5). In models of glomerulosclerosis and in human DKD, endothelial dysfunction has recently been recognized to play a critical role in the initiation and progression of glomerular disease (6, 7). Glomerular endothelial cells (GECs) are in direct contact with substances in the blood and, compared to other renal intrinsic cells, rendering them particularly vulnerable to hyperglycemia and other circulating metabolites (8).

Hyperglycemia-induced vascular endothelial dysfunction involves a complex regulatory process, including defective endothelial repair, insufficient angiogenic capacity, oxidative stress, inflammation, and metabolic abnormalities (9, 10). Elevated intracellular glucose levels lead to endothelial metabolic disturbances and subsequent excessive production of reactive oxygen species (ROS) (11), activating the NF-κB signaling pathway and resulting in the release of vascular cell adhesion molecule-1 (VCAM-1), intracellular adhesion molecule-1 (ICAM-1), among others (12). Dysregulated angiogenesis is also a hallmark of DKD, and it may be attributed to the activation of vascular endothelial growth factor (VEGF) and other pro-angiogenic factors in the diabetic microenvironment (13). Although glomerular endothelial injury is known to precede podocyte injury in various kidney diseases (14), existing research lacks mechanism studies guided by endothelial cell injury. This research bias has left the dynamic interplay between endothelial cells and neighboring cells (podocytes, mesangial cells, tubular epithelial cells) as a major knowledge gap in the understanding of DKD mechanisms. Cellular communication, also known as cellular crosstalk, is typically facilitated through direct cell-to-cell contact or the transfer of secreted molecules and extracellular vesicles (EVs) (15). The kidney is a multicellular organ composed of more than 20 cell types, and these diverse cell types interact both under normal conditions and in response to hyperglycemia (16). Under normal physiological conditions, most intrinsic cells within the kidney, except for epithelial cells, remain relatively quiescent, collectively maintaining the normal structure and physiological function of the kidney. However, in diabetes, stressors including hyperglycemia, advanced glycation end-products (AGEs), and dyslipidemia drive phenotypic alterations, numerical changes, and surface marker dysregulation in resident renal cells (17).

This review discusses the mechanisms by which GECs contribute to endothelial dysfunction in DKD and integrates recent evidence to reveal the multicellular crosstalk network involving GECs, podocytes, mesangial cells (MCs), and renal tubular epithelial cells (TECs), thereby aiding in deepening the understanding of the mechanisms of endothelial dysfunction in DKD from cellular and molecular biology perspectives. Furthermore, this article summarizes therapeutic approaches targeting endothelial function for DKD treatment, including direct glucose-lowering therapies, non-antihyperglycemic interventions, and interventions targeting endothelial injury biomarkers, providing the latest insights for precisely outlining potential interventional strategies.

## 2 Hemodynamics and endothelial function in the kidney

### 2.1 Normal renal microcirculation and glomerular filtration

The human kidney is responsible for maintaining fluid and electrolyte balance, removing metabolic waste, and regulating blood pressure. In healthy adults, the kidneys filter approximately 180 liters of plasma into the nephrons daily. The human kidney contains about 1 million nephrons, each composed of a renal corpuscle (containing the glomerulus) located in the renal cortex and renal tubules that extend throughout all regions of the kidney (18). Based on the anatomical location where the U-shaped loop (i.e., the loop of Henle) turns back, nephrons are classified into three categories: cortical nephrons, short-looped nephrons, and long-looped nephrons, with their turning points located in the cortex, medulla, and papilla, respectively (19, 20). The thick ascending limb (TAL) of the loop of Henle plays a crucial role in major kidney functions. The fluid composition in the late TAL lumen may influence the tubuloglomerular feedback system and thus the GFR (20).

Early DKD is characterized by supraphysiological glomerular hyperfiltration. This early clinical condition is known as glomerular hyperfiltration and results from alterations in both structural and dynamic determinants of GFR caused by obesity and diabetes (21). The glomerulus is a capillary network located within Bowman’s capsule, acting as the kidney’s filtration unit. It ensures selective ultrafiltration by retaining essential plasma proteins from the blood. It consists of fenestrated endothelium, the glomerular basement membrane (GBM), and podocytes, which together constitute the glomerular filtration assembly (22). Podocytes wrap around the glomerular capillaries, interdigitate, and are connected by slit diaphragms bridging the filtration slits. Intraglomerular mesangial cells remain in close contact with the glomerular endothelium, which lies on the side of the GBM opposite the podocytes. The specialized endothelial cells lining the glomerular capillary loops are flat and fenestrated, allowing the high flux required for filtration (23). Furthermore, two other aspects of the mechanism of glomerular hyperfiltration in DKD are: one refers to any process that enhances proximal tubule glucose reabsorption, including the upregulation of sodium-glucose cotransporters (SGLTs) and tubular hyperplasia, subsequently altering GFR via TGF; the other is the elevated levels of certain hormones and vasoactive substances in diabetic patients, which modulate the constriction and dilation of pre- and post-glomerular arterioles, altering vascular resistance and thereby initiating hyperfiltration (24).

In summary, the maintenance of GFR relies not only on the glomerular filtration barrier itself but also on the regulation of glomerular arterioles by TGF and vasoactive substances. Increased glomerular capillary pressure, glomerular enlargement, and disturbed tubuloglomerular feedback are the main causes leading to glomerular hyperfiltration, subsequently resulting in basement membrane expansion, podocyte injury, and increased albuminuria (25).

### 2.2 Endothelial cells and renal endothelial dysfunction

The kidney is a highly vascularized organ. The renal artery progressively branches into interlobar arteries, arcuate arteries, and interlobular arteries, which further branch into smaller afferent arterioles that ultimately lead to the glomerular capillaries, where fluid and solutes (excluding plasma proteins) are filtered. Subsequently, the other end of the glomerular capillaries forms the efferent arterioles, which enter the peritubular capillary network (26). Afferent arterioles primarily arise from interlobular arteries and supply blood to the glomerular capillaries, forming the first capillary network within the kidney; glomerular blood flow is drained via efferent arterioles; in the cortical region, efferent arterioles branch to form peritubular capillaries, which surround the cortical tubules, collectively constituting the second capillary network (27). The glomerular capillary network is primarily responsible for filtering plasma to form the primary urine. Endothelial cells in this network share the basement membrane with podocytes and collectively build the filtration barrier through unique intercellular interactions (23). The homeostasis of renal endothelial cells is crucial for preserving glomerular structure and function, maintaining an anti-inflammatory and anti-thrombotic environment, and preventing renal fibrosis (28).

Under diabetic conditions, persistent hyperglycemia directly damages endothelial cells by promoting the generation of advanced glycation end products (AGEs), activating the RAGE receptor, and inducing oxidative stress and inflammation (9). Endothelial cells are both targets and sources of vasoactive substances (such as nitric oxide, prostacyclin, angiotensin II, endothelin-1, etc.). The combined effect of abnormal endothelial vasodilatory responses and imbalances in substances regulating vascular tone leads to endothelial dysfunction (29). Endothelial dysfunction and progressive rarefaction of the renal microvasculature are central drivers of kidney injury associated with major conditions such as hypertension, dyslipidemia, and diabetes (30).

Endothelial dysfunction directly participates in and exacerbates two core pathophysiological processes in DKD: Firstly, hyperglycemia increases the filtered glucose load in the proximal tubules of diabetic patients. This leads to overactivity of SGLT2 and SGLT1, thereby increasing tubular reabsorption of glucose and sodium, and reducing solute delivery to the macula densa, leading to downstream activation of TGF (31, 32). This increased proximal sodium reabsorption reduces ATP breakdown and adenosine production. Adenosine is a potent vasoconstrictor, and its reduction leads to afferent arteriolar vasodilation, consequently causing hyperfiltration (33) On the other hand, early after weight gain, renal vasodilation, increased renal blood flow (RBF), and glomerular hyperfiltration occur (34). Brenner’s research found that glomerular hyperfiltration accompanied by glomerular capillary hypertension is the pathological mechanism for the initiation and progression of nephropathy (35). Secondly, the persistence of endothelial dysfunction progresses from functional impairment to structural damage. Studies have found that hyperglycemia can lead to thickening of the glomerular basement membrane (36). Over time, these injuries may become irreversible, leading to capillary rarefaction (37). Aberrant renal capillary formation may lead to glomerular morphological changes and inflammatory cell infiltration (38). Capillary endothelial injury and subsequent capillary rarefaction can accelerate glomerulosclerosis and tubulointerstitial fibrosis in all types of kidney diseases (39, 40). Renal fibrosis is the common endpoint of all progressive chronic kidney diseases, also indicating that kidney injury has entered an advanced stage (41).

## 3 Pathogenesis of renal cell injury in endothelial dysfunction in DKD

### 3.1 Glomerular endothelial cells

GECs are divided into three subtypes: afferent arteriole-associated glomerular renal endothelial cells (gRECs), efferent arteriole-associated gRECs, and Capillary gRECs. Among them, gRECs in the upstream (most distal) part of the afferent arterioles express genes involved in vasotone regulation such as Edn1 (which encodes endothelin 1), arachidonate 12-lipoxygenase (Alox12) and sphingosine-1-phosphate receptor 1 (S1pr1). And gRECs in the efferent arterioles show lower connexin expression, especially connexin 37 and connexin 40 (encoded by Gja4 and Gja5, respectively). Capillary gRECs possess non-diaphragmed fenestrations that allow the filtration of high volumes of fluid. Capillary gRECs also produce the glycocalyx layer composed of negatively charged glycoproteins and polysaccharides. And Capillary gRECs synthesize and share the glomerular basement membrane (GBM) with podocytes. Capillary gRECs exhibit various genes involved in glomerular capillary formation, including Endoglin (Eng), SMAD Family Member 6 (Smad6), Smad7, X-Linked Inhibitor of Apoptosis (Xiap) and Homeodomain-Interacting Protein Kinase 2 (Hipk2) (42). Although direct evidence elucidating the differential responses of different GECs subtypes to hyperglycemia is currently lacking, the heterogeneity in their anatomical locations, functional characteristics, and gene expression suggests that different subtypes likely respond to hyperglycemia through specific mechanisms. Furthermore, during the progression of DKD, the mechanisms of GECs injury have different emphases.

The early stage of diabetic kidney disease is characterized by neoangiogenesis and increased vascular permeability (43). In the early course of the disease, intracellular hyperglycemia leads to abnormal blood flow and increased vascular permeability due to reduced activity of vasodilators such as nitric oxide (NO), increased activity of vasoconstrictors like angiotensin II and endothelin-1, and alterations in permeability factors such as vascular endothelial growth factor (VEGF) (44). GECs constitute a vital component of the glomerular filtration barrier, maintaining vascular permeability through their fenestrated structure and glycocalyx coating (45, 46). Hyperglycemia promotes vascular dysfunction by degrading the glycocalyx (47), and glycocalyx shedding along with increased markers of oxidative stress indicate impaired endothelial function (48). Singh et al. (49)demonstrated that glycocalyx injury is involved in the pathogenesis of diabetic proteinuria; under high glucose (HG) conditions, Heparan sulfate glycosaminoglycan (HS-GAG) was significantly reduced in cultured GECs, and albumin passage across the GECs monolayer was significantly increased, an effect replicated by enzymatic removal of HS from the GECs glycocalyx. Junlin Zhang et al. (50)found that ABCA1 deficiency led to decreased expression of the tight junction-associated protein ZO-1 and the glycocalyx core proteins syndecan-1 and glypican-1 in GECs, increased ET-1 expression, and induced endothelial glycocalyx injury. Fan Yi et al. (51)discovered that Homocysteine (Hcys) induced significant increases in serine phosphorylation of p47phox and its translocation to the cell membrane in GECs, enhanced ceramide production in GECs, triggered the clustering of lipid rafts (LRs) on the GECs membrane, and formed redox signaling platforms by aggregating and activating NADPH oxidase subunits, thereby enhancing superoxide (O_2_^.−^) production, inducing disruption of the GECs microtubule network, and increasing GECs permeability.

Besides glycocalyx injury and altered vascular permeability, mechanisms related to hormones and insulin sensitivity also contribute to GECs dysfunction. Studies indicate that the local renin-angiotensin system (RAS) in GECs is activated under HG conditions, leading to elevated angiotensin II (AII) levels, which increases the width of glomerular endothelial cell fenestrae, contributing to the development of albuminuria in DKD (52). Aminophospholipids (lipid-AGEs) can affect GECs function by interfering with the insulin signaling pathway, specifically by enhancing the inhibitory phosphorylation of Insulin Receptor Substrate-1 (IRS1) in GECs, activating AKT (protein kinase B) and p42/p44 ERK, and leading to aberrant angiogenesis (53).

NO controls vascular tone and regulates endothelial inflammation and coagulation properties. NO deficiency is a core aspect of endothelial dysfunction, and reduced NO bioavailability may result from altered activity of endothelial nitric oxide synthase (eNOS) or accelerated NO removal (30). eNOS is expressed in GECs and continuously produces NO under physiological conditions, playing a protective role against glomerular injury (54). However, the high glucose environment can induce GECs dysfunction, manifested as increased apoptosis, reduced eNOS dimerization, impaired phosphorylation of eNOS at serine 1179, and ultimately decreased NO synthesis (55). The role of eNOS loss-of-function in DKD progression is further supported by evidence showing that diabetic eNOS-knockout mouse models (STZ eNOS−/−) exhibit more severe apoptosis, oxidative stress, and abnormal vascular proliferation compared to streptozotocin-induced diabetic wild-type mice. Furthermore, STZ eNOS−/− mice display a biphasic change in GECs number, characterized by an initial increase followed by a decrease over time, ultimately leading to more GECs death during DKD progression (56). Research found (57)that asymmetric dimethylarginine (ADMA), an endogenous eNOS inhibitor, reduces the expression of the endothelial marker FLK-1 (VEGFR2, Vascular Endothelial Growth Factor Receptor 2) while upregulating the expression of fibronectin, transforming growth factor-β (TGF-β), and connective tissue growth factor (CTGF), promoting the transition of endothelial cells towards a mesenchymal phenotype. In animal models, pharmacological inhibition of NO synthesis also significantly reduced capillary density in the renal medulla, confirming that NO deficiency leads to capillary rarefaction.

Notably, the role of NO in DKD is bidirectional: both insufficient and excessive levels can impair endothelial function. One study found (58)that acute hyperglycemia upregulated Piezo1 expression in mouse kidneys, and cell experiments revealed that hyperglycemia upregulated Piezo1 expression and the expression/phosphorylation of p-CaMKII (phosphorylated Ca2+/Calmodulin-dependent protein kinase type II), Akt phosphorylation, and p-eNOS (phosphorylated eNOS), increasing NO production. Piezo1, via CaMKII-mediated eNOS activation, induces hyperosmolarity-driven endothelial vasodilation during hyperglycemia, disrupts the medullary osmotic gradient, leading to excessive fluid and sodium excretion, and increases GFR in mice.

In the transitional phase, mesangial expansion and sclerosis accompany excessive extracellular matrix (ECM) production, gradually leading to renal fibrosis (59). The high glucose environment activates Protein Kinase C β (PKCβ) expression in GECs. PKCβ upregulates swiprosin-1 expression, and Swiprosin-1 promotes GECs apoptosis in DKD via the mitochondrial pathway, leading to significantly increased glomerulosclerosis and ECM deposition, as well as significantly increased urinary albumin in DKD mice (60). Studies show that high glucose culture medium leads to increased expression of Insulin-like growth factor-binding protein 5 (IGFBP5) in endothelial cells. IGFBP5 activates the expression of 6-phosphofructose-2-kinase/fructose-2,6-bisphosphatase 3 (PFKFB3) via EGR1, enhances ECM production, thereby promoting the production of pro-inflammatory molecules including interleukin-6 (IL-6), TNF-α (tumor necrosis factor-α), the chemokine monocyte chemoattractant protein-1 (MCP-1), and the adhesion molecule intercellular adhesion molecule-1 (ICAM-1), increases macrophage migration, promotes glycolysis and inflammatory responses in ECs, enhances renal inflammation, and promotes DKD progression. In contrast, mice with PFKFB3 knockout showed significantly attenuated development of glomerular hypertrophy and mesangial expansion (61).

Renal fibrosis is a prominent pathological feature of the late stage of DKD (59). Under pathological conditions, GECs undergo endothelial-mesenchymal transition (EndMT), leading to reduced glomerular capillary density, loss of glomerular endothelial permeability, and ultimately resulting in renal fibrosis and dysfunction (62, 63). Studies indicate that multiple mechanisms, including AGEs deposition, transforming growth factor-β (TGFβ) activation, platelet-derived growth factor B (PDGF-B), Angiotensin II (AngII, Ang2), and microRNAs, participate in the occurrence of EndMT in GECs during DKD (64). The high glucose environment significantly reduces the expression of endothelial markers CD31 and VE-cadherin in GECs, significantly increases the expression of fibroblast-like markers such as α-SMA, markedly enhances the migratory capacity of GECs, and induces the occurrence of EndMT (65).

Quan Hong et al. (66)found that HG conditions induce leucine rich alpha-2-glycoprotein 1 (LRG1) expression in mGECs. LRG1 can enhance ALK1-mediated angiogenesis, thereby promoting DKD progression. Xuan Wang et al. (67)examined the effects of LRG1 deletion in renal cells of diabetic OVE26 mice using single-cell transcriptomics and found that inhibiting Lrg1 led to significant reversal of genes within the angiogenesis and TGF-β pathways. The most widely recognized gene expression changes induced by TGF-β signaling are related to pro-fibrotic genes associated with ECM synthesis and deposition. LRG1 deletion also normalized the expression of TGF-β pathway-related genes in podocytes and mesangial cells, alleviating diabetic glomerulopathy. Lipid environment stimuli such as oxidized LDL (ox-LDL) can also stimulate increased expression of TGF-β and fibronectin protein in GECs, promoting glomerulosclerosis (68).

**Figure 1:**
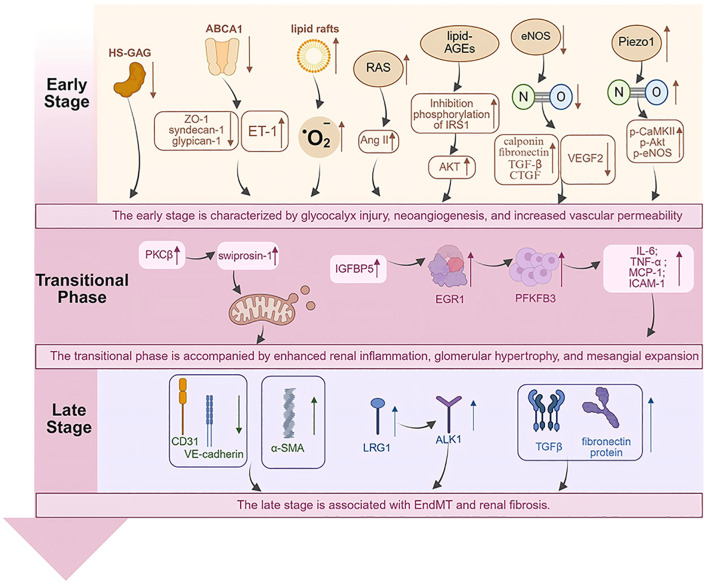
Pathogenesis of GECs injury in endothelial dysfunction in DKD.

In summary, injury to GECs plays a central role in driving the pathological progression of DKD, culminating in the deterioration of renal function from early-stage dysfunction to end-stage failure. The early stage is characterized by glycocalyx injury, neoangiogenesis, and increased vascular permeability. The transitional phase is accompanied by enhanced renal inflammation, glomerular hypertrophy, and mesangial expansion. The late stage is associated with EndMT and renal fibrosiss (**Figure 1**).

Endothelial dysfunction in DKD evolves through three stages. The early stage features glycocalyx damage and hyperpermeability, primarily due to aberrant glycocalyx protein expression, oxidative stress, and impaired eNOS function. The transitional phase is defined by inflammatory activation leading to glomerular hypertrophy and mesangial expansion, predominantly through PKCβ-Swiprosin-1 pathway activation and upregulation of inflammatory factors. The late stage is dominated by EndMT-mediated fibrosis, characterized by downregulation of CD31 and VE-cadherin, upregulation of α-SMA, and renal fibrosis orchestrated by the LRG1-ALK1 and TGFβ signaling axes. Created in https://BioRender.com.

### 3.2 Crosstalk between GECs and other cells

#### 3.2.1 Podocytes and GECs

The glomerular filtration barrier (GFB) consists of three layers. The first layer is built of specialized glomerular endothelial cells characterized by a high number of perforations called fenestrae. The second layer is composed of collagen IV, laminins, fibronectins, and proteoglycans organized in a GBM. The last layer is formed by podocytes with interdigitating foot processes that are bridged by slit diaphragms (69, 70). Podocytes possess a unique morphology, with each podocyte simultaneously supporting multiple capillary loops (71). Glomerular endothelial cells synthesize and secrete various cytokines to regulate glomerular hemodynamics and interactions with other renal intrinsic cells (17). In models of primary podocyte-restricted injury, glomerular endothelial cells exhibit mitochondrial oxidative stress (mtStress) associated with endothelial dysfunction, which is essential and precedes podocyte apoptosis and loss (72).

##### 3.2.1.1 Autophagy and co-culture systems

Autophagy is a key protective mechanism for both the endothelial cell and podocyte layers of the GFB. Olivia Lenoir et al. found that short-term high glucose conditions can induce the accumulation of Microtubule-associated protein 1 light chain 3B-II (LC3B-II) in podocytes, triggering podocyte autophagy. Specific deletion of Autophagy-related protein 5 (Atg5) in podocytes led to diabetes-induced GBM thickening, and widening and effacement of podocyte foot processes. On the other hand, specific deletion of Atg5 in vascular endothelial cells induced mild morphological lesions in the GFB, such as loss of endothelial fenestrae. Loss of autophagy in both cell types exacerbated DKD by disrupting GFB integrity, suggesting a synergistic maintenance of glomerular homeostasis (73). Co-culture experiments of podocytes and GECs revealed that the diabetic environment causes stress in GECs, leading to the secretion of pro-apoptotic factors like caspase 3 into the supernatant affecting podocytes, causing a significant increase in podocyte apoptosis (74). Michael Albrecht et al. (75) stablished a co-culture system where GECs and podocytes grow closely together. The results showed that under co-culture conditions, HG promoted significant upregulation of Early Growth Response 1 (EGR1), Nuclear Receptor Subfamily 4 Group A Member 1 (NR4A1), C-X-C Motif Chemokine Ligand 1 (CXCL1), and Colony Stimulating Factor 2 (CSF2). When GECs were cultured independently without podocytes, none of these genes were upregulated by HG, indicating that interaction with podocytes helps control the gene expression in GECs in response to HG.

##### 3.2.1.2 Bidirectional regulation of VEGF signaling

Members of the VEGF family, including Vascular endothelial growth factor A (VEGF-A), VEGFB, VEGFD, VEGFE, and placental growth factor, are key regulators of endothelial cell function (76). In the kidney, podocyte-derived VEGF-A is crucial for maintaining the structure and function of glomerular capillaries (77). It can bind to two different tyrosine kinase receptors on vascular endothelial cells, VEGF receptor 1 (VEGFR1) and VEGFR2, regulating angiogenesis and vascular permeability (16, 78). Some studies suggest that elevated VEGF-A levels are responsible for GECs dysfunction and albuminuria in DKD, and that VEGF-C can counteract these effects of VEGF-A. Researchers first locally induced VEGF-C overexpression in podocytes of otherwise healthy mice and found that VEGF-C could reduce VEGF-A mRNA expression without adverse effects on glomerular function. However, in a type 1 diabetes model, induction of VEGF-C overexpression in podocytes was shown to attenuate the loss of glomerular endothelial fenestrations (79).

Other studies suggest that both selective deletion and overexpression of VEGF-A in the glomerulus can cause glomerular disease in mice. In mice with podocyte-specific overexpression of VEGF-A, collapse of glomerular capillary loops towards the vascular pole, enlargement of Bowman’s capsule, and increased endothelial cell numbers were observed, suggesting that excessive VEGF drives glomerular structural disruption. When podocyte-specific VEGF-A was knocked out, a significant reduction in endothelial cell number and lack of fenestrae were observed, indicating that insufficient VEGF levels also directly damage the endothelium (80).

##### 3.2.1.3 Synergistic regulation of the angiopoietin/Tie-2 system

Furthermore, the angiopoietin (Angpt) family also plays an important role in renal angiogenesis, primarily consisting of Angpt1-4 (Ang1-4) (16). Ang1 is produced by podocytes and vascular support cells (such as pericytes), while Ang2 is produced and released from Weibel-Palade bodies in endothelial cells (ECs) under stress (81, 82) The angiopoietins Ang-1 and Ang-2 have been identified as ligands of the receptor tyrosine kinase Tie-2 (83) The Ang/Tie-2 signaling pathway plays an important role in vascular development and function. Tie-2 is a receptor tyrosine kinase specifically expressed in endothelial cells. Ang1 can act as a Tie-2 agonist promoting angiogenesis (84). Ang2 signals in an endocrine or autocrine manner; it also binds Tie-2 but can act as either an agonist or antagonist of Tie-2. Elevated Ang2 levels are associated with adverse outcomes in vascular disease (85). Induction of podocyte-specific Ang2 overexpression in mice led to more proteinuria and GECs apoptosis, which is detrimental to GECs integrity and filtration barrier function (86). Furthermore, restoration of podocyte-specific Ang1 expression in DM mice led to increased Tie2 phosphorylation in the renal cortex and improved GECs proliferation (87).

##### 3.2.1.4 Other key crosstalk factors

Endothelin-1 (EDN1, ET1) is an endothelial-derived polypeptide with potent vasoconstrictive effects (88). Previous studies have shown that ET-1 can activate NF-κB, MAPK, and other signaling pathways, leading to mesangial matrix expansion, glomerulosclerosis, and podocyte injury (89)ET-1 induces a series of pathophysiological changes by binding to the ETA receptor (ETAR) and ETB receptor (ETBR) (90). Ilse Daehn et al. (72)found that activation of TGFβR1 in podocytes leads to SMAD2/3-mediated synthesis and release of EDN1 and induces upregulation of EDNRA expression in GECs. Podocyte-derived EDN1 acts on endothelial cells, inducing mitochondrial ROS and endothelial dysfunction while reducing NO synthesis. Another study showed that podocyte-derived EDN1, through EDNRA and mitochondrial ROS, increases the levels of heparanase (Hpse) and hyaluronidase (Hyal) in GECs, inducing degradation and remodeling of the ESL (91).

Growing evidence suggests that Krüppel-like factors (KLFs) are key regulators of endothelial biology. In particular, KLF2 plays an important role in regulating endothelial function (92). Zhong et al. observed decreased KLF2 levels in the glomeruli of DM rats and in renal tissues of DKD patients. Furthermore, they demonstrated that specific knockout of KLF2 in GECs of diabetic mice led to more severe kidney injury, particularly severe podocyte and GECs injury, such as reduction of podocyte differentiation markers like synaptopodin, podocin, and podocalyxin, causing foot process effacement and reduced podocyte number. This suggests that KLF2 derived from GECs may be a potentially important molecule in podocyte injury (93).

Semaphorin 3A (Sema3A) is primarily produced by mature podocytes. Sema3A deficiency leads to abnormal renal vascular morphology, excessive glomerular endothelium, widened podocyte foot processes, and proteinuria. However, overexpression of podocyte Sema3A during organogenesis causes glomerular hypoplasia, characterized by endothelial cell apoptosis (94).

The high glucose environment prompts GECs to secrete more exosomes. The increased exosomes from these EndMT-undergoing cells can increase TGF-β1 expression in podocytes and induce Wnt1 mRNA expression in podocytes, activating Wnt/β-catenin signaling, inducing podocyte epithelial-mesenchymal transition (EMT) and dysfunction, disrupting filtration barrier integrity, and ultimately leading to albumin leakage (95).

#### 3.2.2 Mesangial cells and GECs

MCs have a low cytoplasm-to-nucleus ratio and contain fibrils in their cytoplasm, supporting glomerular structure and regulating glomerular filtration. Furthermore, MCs regulate the balance between mesangial matrix production and degradation and signaling with other cells to maintain normal glomerular homeostasis (96) The area without direct contact between MCs and the endothelial wall is called the extraglomerular mesangium (97). The extraglomerular mesangium separates MCs and the GBM via ECM secreted by MCs (98). MCs can proliferate and expand to contact the inner layer of the GBM, leading to capillary collapse when capillaries separate from the GBM (99). Furthermore, due to mesangial expansion, the proportion of MCs in the glomerulus increases, ECM increases, obstructing glomerular capillaries and leading to glomerulosclerosis (100). MCs, as stromal cells, are integral components of glomerular structure, maintaining the homeostasis of GECs and podocytes (101). MCs and GECs are closely coupled and MCs control the surface area of glomerular capillaries through relaxation and contraction, thereby influencing GFR (102).

##### 3.2.2.1 Oxidative stress and exosomal communication

The NADPH oxidase (NOX) family is a major source of reactive oxygen species (ROS) in the vasculature and can regulate renal perfusion (103). Endothelial NAD(P)H oxidase activation accelerates early glomerular injury in diabetic mice by increasing ROS. Hajime Nagasu et al. (104) found that in the kidneys of diabetic mice with endothelial-targeted overexpression of the catalytic subunit of NAD(P)H oxidase, Nox2 (NOX2TG), endothelial cell ROS levels were elevated, the α-SMA stained area in mesangial cells was enlarged, endothelial cells exhibited numerous swollen vesicles and loss of capillary fenestrae, the endothelial glycocalyx was reduced, and glomerular capillary permeability was increased. Li Ling et al. (105) demonstrated that exosomes rich in circular RNA (circRNA) released from HG-treated GECs promoted the expression of α-smooth muscle actin (α-SMA) in glomerular mesangial cells (GMCs) and induced EMT in MCs.

##### 3.2.2.2 TGF-β signaling pathway

The TGF-β signaling pathway plays a crucial role in renal fibrosis in diabetic nephropathy. TGF-β is a key mediator of fibrosis, exerting its pro-fibrotic effects by activating downstream signaling, leading to EMT, End-MT, and myofibroblast activation (106). Compared to GECs treated with normal glucose (NG), HG-treated GECs secreted more glomerular endothelial exosomes containing TGF-β1 mRNA. These exosomes are taken up by MCs and mediate MC activation via the TGF-β1/SMADs signaling pathway. This activation leads to significant mesangial expansion, proliferation, and excessive production of ECM proteins (105, 107). Hiroaki Tsuruta et al. (108) reported that Lrg1 protein derived from GECs may translocate to the mesangial region, exacerbating glomerulosclerosis through upregulation of TGF-β signaling and increasing fibrosis by upregulating Plasminogen activator inhibitor-1 (PAI-1) gene expression (a pro-fibrotic marker) via the p38 pathway in mesangial cells. Specifically, TGF-β1 phosphorylated SMAD2 and SMAD3 without affecting p38 phosphorylation, whereas Lrg1 enhanced p38 phosphorylation without affecting SMAD2 or SMAD3 phosphorylation. Enhanced PAI-1 expression by Lrg1 in the presence of TGF-β1 was also observed under high glucose conditions. These findings indicate that SMAD pathway activation is essential for TGF-β1-induced PAI-1 expression, and Lrg1-mediated p38 activation may enhance the SMAD pathway and subsequent PAI-1 expression upon TGF-β1 stimulation, accelerating glomerulosclerosis and fibrosis. They reported a novel intracellular crosstalk termed “endothelial cell-mesangial cell (EC-MEs) crosstalk”.

##### 3.2.2.3 Other key crosstalk factors

Integrin αvβ8 is most abundantly expressed in the kidney and brain, and its primary ligand is latent transforming growth factor-β (TGF-β). Renal αvβ8 is localized to mesangial cells. Itgb8−/− mesangial cells exhibited reduced latent TGF-β binding, leading to bioactive TGF-β release, which stimulated GECs apoptosis (109).

Yi-Chun Tsai et al. (110) used scRNA-seq to study early changes in the kidneys of db/m and db/db mice at week 14. The results showed that semaphorin 3C (SEMA3C) secreted by MCs induced GECs injury via Neuropilin 1 (NRP1)/Neuropilin 2 (NRP2), modulating crosstalk in the early DKD glomerular microenvironment and promoting endothelial-mesenchymal transition in glomerular endothelial cells. Blocking NRP1 or NRP2 ameliorated End-MT and the increased GECs permeability induced by HG-stimulated MCs supernatant. Similarly, inhibiting NRP1 or NRP2 attenuated SEMA3C-induced GECs injury, including End-MT and increased permeability.

#### 3.2.3 Renal tubular epithelial cells and GECs

TECs line multiple different nephron segments beyond the glomerulus, facing the remarkable task of converting 180 liters of glomerular filtrate into 1–2 liters of urine daily. Beyond simple solute and water reabsorption, nephron epithelial cells respond to and control the overall organismal balance of acids, solutes, fluids, hormones, vitamins, and xenobiotics (111). TECs are resident cells of the tubulointerstitium and play important roles in kidney recovery after injury and in renal fibrosis (112).

##### 3.2.3.1 Metabolic coupling and inflammation

Proximal Tubular Epithelial Cells (PTECs) are known for their high energy consumption, possessing abundant mitochondria, peroxisomes, and other metabolic organelles (113). Under glucotoxic conditions, a metabolic shift from mitochondrial oxidative metabolism to glycolysis supports TECs proliferation and differentiation, thereby promoting EMT occurrence (114). An association exists between fatty acid oxidation (FAO) and oxidative phosphorylation (OXPHOS) in proximal tubule (PT) cells of patients with kidney disease and PT cell differentiation and disease. Studies found that FAO and OXPHOS directly drive PT cell differentiation, representing how metabolism can drive cell state (115). DKD is considered an inflammatory disease caused by disorders of glucose and lipid metabolism. High concentrations of urinary albumin in DKD patients activate TECs, producing pro-inflammatory factors such as CRP, TNF-α, NF-κB, and ROS, which may cause GECs injury, apoptosis, and EndMT (116). The close proximity between endothelial and epithelial cells allows TNF-α released by endothelial cells to stimulate epithelial cells to release IL-6 and IL-8, thereby amplifying the inflammatory process in the proximal tubule (117). The glomerular vascular network undergoes apoptosis, necrosis, and transdifferentiation under the stimulation of inflammatory factors, disrupting the structure and function of GECs. Damaged GECs reduce the blood supply to the tubules, exacerbating TECs injury (46).

##### 3.2.3.2 Hypoxia signaling pathway

In the pathological process of diabetic nephropathy, hypoxia can activate hypoxia-inducible factor-alpha (HIF-α) in renal tubular epithelial cells, which in turn induces high expression of Metallothionein 3 (MT3). MT3 then inhibits HIF-1α activity through a negative feedback mechanism, leading to reduced downstream VEGF expression, ultimately causing glomerular endothelial cell swelling (118). Another study found that endothelial microparticles (EMPs) released by endothelial cells, by upregulating COX-2 expression, increase the expression of HIF-α and VEGF-A in human renal proximal tubule HK-2 cells, contributing to the pathogenesis of kidney disease (119). VEGF-A is expressed by specific epithelial cells along the nephron and communicates with its receptor VEGFR2 on peritubular capillary endothelial cells to maintain the development and density of the peritubular capillary network; its specific deletion leads to reduced capillary density and increased renal erythropoietin (Epo) production (120).

##### 3.2.3.3 Extracellular vesicle-mediated information transfer

Extracellular vesicles (EVs) are emerging as new mediators of intercellular crosstalk, capable of delivering epigenetic regulators to proximal or distal recipient cells and influencing target cell behavior (121). Under ischemia/reperfusion (I/R) injury, TECs shift the secretion pattern of VEGF-A from a free form to a small extracellular vesicle (sEV)-carried form (sEV-VEGF-A). These sEVs can be taken up by peritubular capillaries (PTCs) endothelial cells in a VEGFR1/2-dependent manner, thereby promoting endothelial cell proliferation, repairing PTC rarefaction, improving renal perfusion, and protecting against the chronic progression of AKI (122), which holds high reference value for DKD research. This discovery provides a new perspective for understanding how TECs instruct endothelial cell repair. However, experimental research directly targeting EV-mediated cross-communication between TECs and GECs in DKD remains limited and is an important direction for future research.

##### 3.2.3.4 Paracrine signaling network

Meanwhile, protective paracrine mechanisms are equally important. Hepatocyte growth factor (HGF) secreted by GECs prevented the overexpression of binding immunoglobulin protein (BiP), C/EBP homologous protein (CHOP), spliced X-box-binding protein 1 (s-XBP1), and interleukin 6 (IL-6) in co-cultured Human Kidney-2 (HK-2) cells exposed to Palmitic acid (PA), alleviating induced endoplasmic reticulum(ER) stress, inflammation, and apoptosis in HK-2 cells in a paracrine manner to reduce kidney injury and exert anti-fibrotic effects (123). A complex communication network exists between microvascular endothelial cells and proximal tubular epithelial cells. Transcriptomic analysis of a co-culture system involving endothelial cells and proximal tubular epithelial cells indicated that HK-2 cells also exhibited an enhanced paracellular gating function in co-culture, which depended on endothelial-derived extracellular matrix. Differentially expressed genes in endothelial cells primarily act as regulators of epithelial cell function, such as extracellular matrix proteins (e.g., collagen I, III, IV, and V, laminin-alpha IV) and cytokines/growth factors (e.g., hepatocyte growth factor, endothelin-1, VEGF-C) (124).

**Figure 2:**
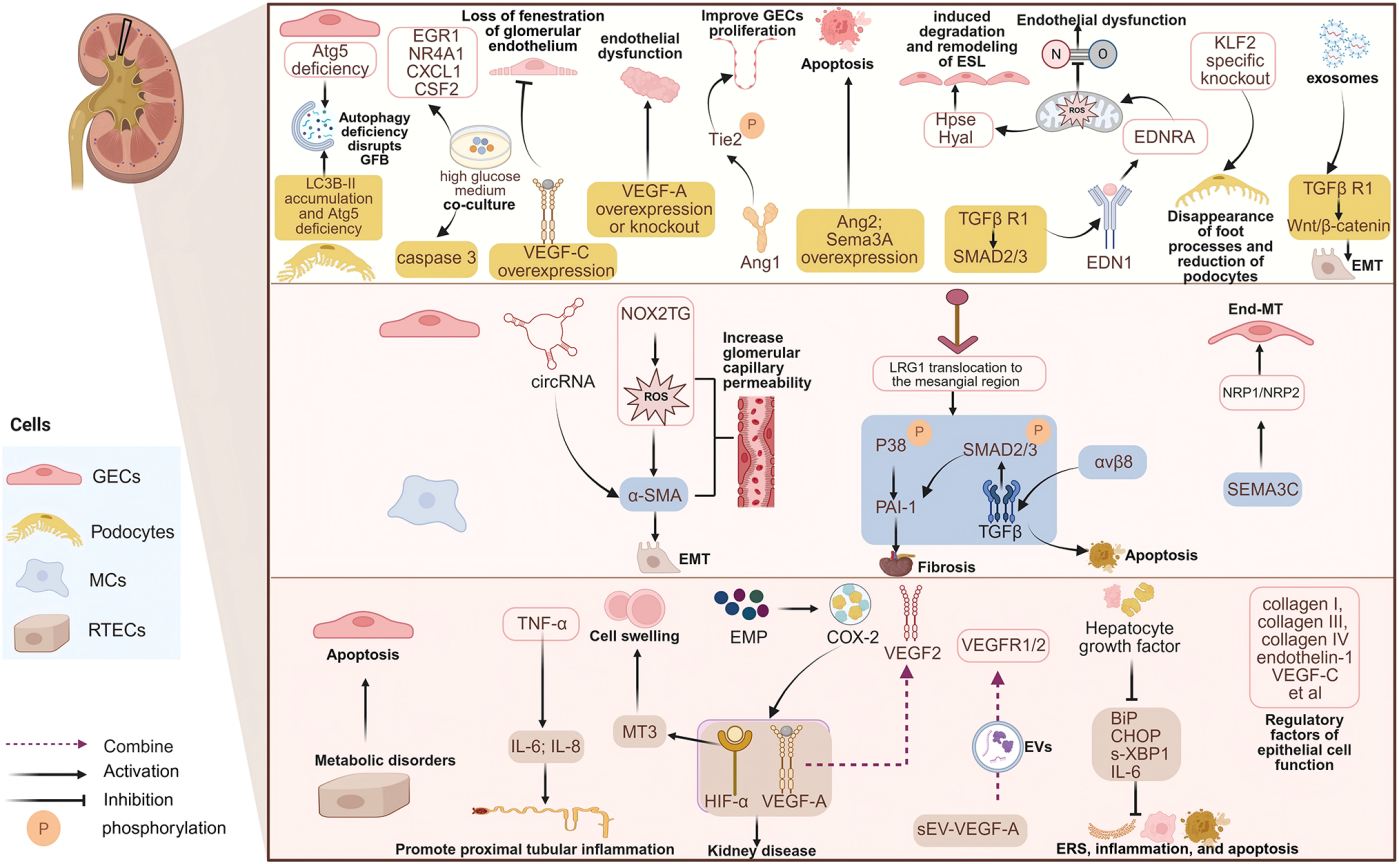
Crosstalk between GECs and other cells in endothelial dysfunction in DKD.

In summary, in DKD endothelial dysfunction, the mechanism of GECS interacting with podocytes, MCs and TECs has different emphasis (**Figure 2**).

Endothelial dysfunction in DKD involves crosstalk between GECs and other cells. GECs and podocytes interact through mechanisms including autophagy, VEGF and Angiopoietin/Tie-2 signaling, ET-1, KLFs, Sema3A, and exosome-driven TGF-β/Wnt/β-catenin activation. Crosstalk with mesangial cells occurs primarily through oxidative stress, exosomal, the LRG1-TGFβ pathway, and SEMA3C-NRP1/NRP2 signaling. Meanwhile, interactions with tubular epithelial cells are mediated by metabolic coupling, inflammatory responses, hypoxia signaling, extracellular vesicles, and paracrine signals. Created in https://BioRender.com.

## 4 Targeting endothelial function for the treatment of diabetic kidney disease

### 4.1 Direct anti-hyperglycemic therapies

#### 4.1.1 Sodium glucose cotransporter 2 inhibitors

SGLT2 inhibitors are oral medications used to reduce hyperglycemia in patients with T2DM, including empagliflozin, ertugliflozin, dapagliflozin, and canagliflozin. They act by inhibiting SGLT2 to reduce renal reabsorption of filtered glucose and lower the renal glucose threshold, thereby increasing urinary glucose excretion (125). SGLT2 inhibition activates tubuloglomerular feedback, leading to afferent arteriolar vasoconstriction and efferent arteriolar vasodilation. These hemodynamic changes initially decrease the GFR but ultimately contribute to the renal protective effects of SGLT2 inhibitors (126). The current understanding of the renoprotective mechanisms of SGLT-2 blockers is based on three potential explanations. First, these drugs, via tubuloglomerular feedback regulation leading to afferent arteriolar vasoconstriction, reduce hyperfiltration and promote glucose excretion through natriuresis; therefore, SGLT-2 blockers exert renal protection through their effects on glomerular hemodynamics. Second, they reduce proximal tubule glucose reabsorption, lower peritubular glucose concentrations, and alleviate edema and fibrosis, protecting renal function. Third, the use of these drugs induces a ketotic state; oxidation of ketones is preferred over free fatty acids, thereby reducing oxidative stress in target organs such as the heart and kidneys (127, 128). Their renal protective effects have been confirmed by large clinical trials. The annual rate of decline in the estimated glomerular filtration rate was slower in the empagliflozin group than in the placebo group (-0.55 vs. -2.28 ml per minute per 1.73m²of body-surface area per year, P<0.001), and empagliflozin-treated patients had a lower risk of serious renal outcomes (129).

Currently, there is a lack of clinical trials directly evaluating the effects of SGLT2 inhibitors on human renal endothelial function in a prospective manner. Supporting evidence mostly comes from preclinical studies. Empagliflozin treatment in DKD mice has been shown to improve renal vascular dysfunction and pathological injury by increasing renal eNOS enzyme activity and upregulating renal NO utilization (130). In BTBR ob/ob mice, empagliflozin was shown to inhibit VEGF-A overexpression in podocytes, increase endothelial cell fenestration density, and reduce the expression of caveolin-1 (CAV-1) and plasmalemma vesicle-associated protein 1 (PV-1), which are associated with glomerular capillary loop formation, thereby inhibiting glomerular endothelial cell remodeling, reducing injury to GECs, restoring the loss of endothelial cell fenestration density, and consequently reducing proteinuria (131).

In summary, the amelioration of renal endothelial function by SGLT2 inhibitors is primarily a secondary effect, mediated through mechanisms such as tubuloglomerular feedback, reduced proximal tubule glucose reabsorption, and podocyte protection, rather than via direct action on GECs.

#### 4.1.2 Glucagon-like peptide-1 receptor agonist

GLP-1 is a hormone released by intestinal L cells after oral intake of glucose and other macronutrients; it promotes insulin secretion and enhances insulin sensitivity in peripheral tissues (132). GLP-1 agonists include liraglutide, lixisenatide, and taspoglutide (in Phase III clinical trials since 2010), as well as drugs that inhibit the primary enzyme degrading GLP-1 [dipeptidyl peptidase-4 (DPP-4) inhibitors], such as sitagliptin, saxagliptin, and linagliptin (133). Konstantinos N Aronis et al. found (133) that GLP-1 promotes angiogenesis in human endothelial cells in a dose-dependent manner via the Akt, Src, and PKC pathways. Inhibition of Akt, Src, or PKC led to the suppression of GLP-1-induced angiogenesis. GLP-1 promoted angiogenesis within a dose range of 200 to 1000 nM, with the maximum effect observed at 500 nM, while 2000 nM did not elicit any significant angiogenic response. Animal studies showed that sitagliptin treatment increased the protein expression of angiogenesis biomarkers (CXCR4, SDF-1α, and VEGF) (134).

However, clinical trial evidence directly targeting renal endothelial function endpoints in DKD patients remains limited. Existing studies mostly infer effects through endothelial markers or hemodynamic parameters. Results from one randomized controlled trial showed that compared to the placebo group, the linagliptin group had significantly increased endothelial markers and functional genes Platelet and endothelial cell adhesion molecule 1 (PECAM1) and VEGF-A, and also showed improvement in pulse wave velocity, a measure of arterial stiffness (135). Clinical trial results from Wenjuan Song et al. showed that Liraglutide was able to reduce body mass index (BMI), waist circumference (WC), low-density lipoprotein cholesterol (LDL), cystatin C (f cystatin), and UACR levels in DKD patients, lower levels of inflammatory factors (TNF-α, IL-6), and improve hemodynamics in DKD patients. Specifically, the treatment group had significantly higher end-diastolic velocity (EDV) in the renal artery and segmental arteries compared to pre-treatment levels and the control group, and both peak systolic velocity (PSV) and EDV in the renal interlobar arteries were significantly higher than pre-treatment levels in the treatment group and the control group (136).

In summary, the improvement of renal endothelial function by GLP-1 receptor agonists is primarily an indirect consequence, mediated through the promotion of insulin secretion, pro-angiogenic effects, and the amelioration of hemodynamic parameters and endothelial biomarkers, rather than a direct action on GECs.

#### 4.1.3 Other drugs

Insulin, by activating the PI3K-Akt signaling pathway, activates its downstream sterol regulatory element-binding protein 1 (SREBP-1). Then, via the PI3K-Akt-SREBP-1 pathway, it stimulates the membrane translocation of Rac1, a member of the GTPase RhoA family, from the cytoplasm to the plasma membrane, inducing endothelial cell migration and tube formation, ultimately promoting microvascular endothelial cell migration and angiogenesis (137).

Ru Yang et al. found that C. paliurus triterpenoids fraction (CPT) treatment significantly reduced blood glucose, microalbumin, BUN, and mesangial expansion levels. Furthermore, CPT improved renal endothelial function by reducing VCAM-1 and ICAM-1 levels and blocking glycocalyx loss. *In vitro*, CPT also alleviated H_2_O_2_-induced endothelial injury. Mechanistically, CPT significantly increased the phosphorylation levels of Akt and eNOS and reduced the expression of ROCK and Arg2 both *in vivo* and *in vitro (*138).

Ethyl eicosapentaenoate (EPA-E) purified from fish oil can reduce inflammatory cytokine levels in glomerulonephritis. Toshinori Yasuzawa et al. found that EPA-E improved mesangial expansion and reduced albuminuria in diabetic mice. The inhibition of EndMT by EPA-E treatment might function through suppressing protein kinase Cβ (PKCβ)/TGF-β/plasminogen activator inhibitor-1 (PAI-1) signaling. Therefore, EPA-E has the potential to confer renal protection by modulating EndMT in DKD (139).

Rutin is a polyphenolic flavonoid that can reduce kidney injury. Xiaoming Wang et al. found that rutin prevents HG-induced disruption of the renal endothelial barrier function by activating Nrf2. This process is achieved by reducing reactive oxygen species (ROS) and subsequently inactivating RhoA/ROCK, thereby effectively preventing hyperpermeability induced by hyperglycemia (140).

### 4.2 Non-antihyperglycemic interventions

Keum-Jin Yang et al. (141) found that the non-purine selective xanthine oxidase (XO) inhibitor febuxostat restored diabetes-induced mesangial matrix expansion, reduced TGF-β1 and Col IV expression, inhibited VEGF/VEGFR1 and VEGFR3, and decreased the protein expression of NOX1, NOX2, and NOX4, as well as the mRNA expression of NOX catalytic subunits NoxO1, p22phox, p47phox, and p67phox. The protective effect of XO inhibition against DKD was attributed to the dephosphorylation of Akt and FoxO3a and enhanced eNOS phosphorylation, thereby reversing renal oxidative stress. In cell experiments, it was found that febuxostat could inhibit VEGF by suppressing the NOX-FoxO3a-eNOS signaling pathway, ameliorate oxidative stress in human GECs, and protect against endothelial cell injury in DKD.

Alejandro R Chade et al. (142) found that statins reduce renal injury in renal artery stenosis (RAS) by restoring angiogenesis and attenuating intrarenal microvascular (IMV) remodeling. Simvastatin in RAS enhanced intrarenal angiogenesis and peri-stenotic arteriogenesis and increased the expression of angiogenic growth factors and hypoxia-inducible factor-1alpha. Furthermore, simvastatin reduced tissue transglutaminase expression and IMV inward remodeling, restored IMV endothelial function, reduced fibrogenic activity, and improved renal function. Long-term simvastatin supplementation promoted angiogenesis *in vivo*, reduced ischemia-induced IMV remodeling, and improved IMV function in the stenotic kidney.

Regarding the potential association of Finerenone with improved endothelial function in DKD, results from a clinical trial showed that Finerenone significantly reduced albuminuria, improved the rate of eGFR decline in DKD patients, and reduced urinary β2MG and NAG levels. In patients with chronic kidney disease and type 2 diabetes, Finerenone reduced the risk of the primary composite kidney outcome (ESKD, a sustained decline in eGFR of ≥40%, or renal death) compared to the placebo group (143).

A randomized controlled trial in DKD patients showed that daily supplementation with 400 mg of Tocotrienol-Rich Vitamin E for 8 months significantly improved eGFR and reduced serum creatinine (144).

### 4.3 Interventions targeting endothelial injury biomarkers

Endothelin (ET) -1 is the most potent endogenous vasoconstrictor produced within the vasculature. In a double-blind, randomized crossover study of 22 patients with proteinuric chronic kidney disease, Neeraj Dhaun et al. found that selective endothelin-A receptor antagonism reduced blood pressure, proteinuria, and arterial stiffness, increased renal blood flow, and lowered renal vascular resistance in patients with proteinuric kidney disease of varying severity. Neeraj Dhaun et al. (145) conducted a randomized, double-blind, three-way crossover study in 27 patients with proteinuric CKD and found that treatment with the ET-A antagonist sitaxentan reduced renal ET-1 production and decreased proteinuria. The reduction in proteinuria with sitaxentan was associated with blood pressure reduction (146).

VEGFA is a potent angiogenic factor that signals through its tyrosine kinase receptor VEGFR2 to promote new vessel sprouting, endothelial migration, proliferation, differentiation, and survival (147). Aberrant angiogenesis leading to leaky, immature vessel formation is a common feature of diabetic retinopathy and diabetic nephropathy, suggesting that elevated VEGFA levels are pathogenic (148). Gavasker A Sivaskandarajah et al. found (149) that DM + VEGF KO mice developed significant proteinuria, glomerular matrix expansion and sclerosis, with enlarged tubules containing protein deposits. Glomeruli from VEGFKO mice also exhibited endothelial cell detachment and capillary loop loss. These results indicate that upregulating VEGFA in the diabetic kidney can protect the microvasculature from injury, and appropriate regulation of VEGFA expression is crucial for healthy glomeruli, providing new therapeutic targets and biomarkers for DKD. Targeting either the renal endothelial VEGFR2 receptor alone or PDGFRβ alone profoundly inhibited microvascular rarefaction, interstitial fibrosis, and inflammation. Targeting endothelial-pericyte crosstalk by inhibiting VEGF receptor signaling attenuated renal microvascular rarefaction and fibrosis (150). The names and key mechanisms of action of drugs targeting endothelial function in the treatment of DKD are summarized in **Table 1**.

**Table 1:** Names of drugs targeting endothelial function to treat DKD and their main mechanisms of action.

Differences	Medication/intervention measures	Main mechanism of action
Direct Anti-hyperglycemic Therapies cotransporter 2	SGLT2 Inhibitors	Reduce glomerular filtration rate (129).
		Increase the activity of renal eNOS enzyme and upregulate the utilization of renal NO (130). Inhibit the overexpression of VEGF-A in podocytes, reduce the expression of CAV-1 and PV-1, and restore the loss of endothelial cell fenestration density (131).
	GLP-1 Receptor Agonist	Promotes angiogenesis in human endothelial cells in a dose-dependent manner via the Akt, Src, and PKC pathways (133). Enhances the protein expression of angiogenic biomarkers (CXCR4, SDF-1α, and VEGF) (134). Increases the expression of endothelial markers and functional genes, PECAM1 and VEGF-A (135). Reduces the levels of inflammatory factors (TNF-α, IL-6) and improves hemodynamics in DKD patients (136).
	Other Drugs	Insulin stimulates membrane translocation of Rac1, a member of the GTPase RhoA family, via the PI3K-Akt-SREBP-1 pathway, promoting microvascular endothelial cell migration and angiogenesis (137).
		CPT reduces the expression of VCAM-1 and ICAM-1 and blocks glycocalyx loss. CPT increases the phosphorylation levels of Akt and eNOS and reduces the expression of ROCK and Arg2 both *in vivo* and *in vitro* (138).
		EPA-E inhibits EndMT by suppressing PKCβ/TGF-β/PAI-1 signaling (139).
		Rutin activates Nrf2, reduces reactive oxygen species (ROS), and subsequently inactivates RhoA/ROCK, preventing hyperglycemia-induced hyperpermeability (140).
Non-Antihyperglycemic Interventions	Febuxostat	Reduces the expression of TGF-β1 and Col IV, inhibits VEGF by suppressing the NOX-FoxO3a-eNOS signaling pathway, and ameliorates oxidative stress in human GECs (141).
	Statins	Restores angiogenesis and attenuates IMV remodeling, increasing the expression of angiogenic growth factors and hypoxia-inducible factor-1alpha (142).
	Finerenone	Reduces albuminuria, improves the rate of eGFR decline, and lowers urinary β2MG and NAG levels (143).
	Tocotrienol	Improves eGFR and reduces serum creatinine (144).
Interventions Targeting Endothelial Injury Biomarkers	ET1	Reduces proteinuria and arterial stiffness, increases renal blood flow, and lowers renal vascular resistance (145).
	VEGF	Inhibits microvascular rarefaction, interstitial fibrosis, and inflammation (149, 150).

## 5 Conclusions

DKD is characterized by a progressive decline in kidney filtration function due to persistent diabetes and is the most common complication of diabetes, affecting approximately 40% of all diabetic patients (151). DKD imposes a high disease burden worldwide and can significantly increase the risk of renal failure and cardiovascular events (152). Endothelial dysfunction of the renal microvasculature, along with functional and structural rarefaction, plays a prominent role in inducing kidney injury associated with hypertension, dyslipidemia, diabetes, and other conditions (30).

Investigating the mechanisms by which GECs lead to endothelial dysfunction and their crosstalk with other renal cells is crucial for better understanding the endothelial dysfunction in DKD and for early intervention in its treatment. As mentioned previously, many studies have reported the characteristics and significant pathogenic roles of GECs function and intrarenal cellular crosstalk in DKD endothelial dysfunction. The heterogeneity of GECs determines the dominant mechanisms at different stages of the disease: the early stage is characterized by glycocalyx injury, neoangiogenesis, and increased vascular permeability; endothelial dysfunction in the transitional phase is accompanied by enhanced renal inflammation, glomerular hypertrophy, and mesangial expansion; the late stage is associated with EndMT and renal fibrosis. In terms of intercellular crosstalk, podocytes and GECs interact synergistically through aspects such as autophagy and co-culture systems, bidirectional regulation of VEGF signaling, and coordinated regulation of the angiopoietin/Tie-2 system. MCs and GECs interact synergistically through aspects such as oxidative stress and exosomal communication, and the TGF-β signaling pathway. TECs and GECs interact synergistically through aspects such as metabolic coupling and inflammation, hypoxia signaling pathways, extracellular vesicle-mediated information transfer, and paracrine signaling networks. This indicates that vascular endothelial dysfunction in DKD arises both from intrinsic dysfunction of GECs themselves and from the mutual interactions between GECs and other cells, including podocytes, MCs, and TECs. Based on this, early diagnosis can focus on the exploration and validation of potential biomarkers, such as Piezo1, VEGF, ET-1, the angiopoietin family, LRG1, and exosomes. The application of these biomarkers holds promise for achieving early identification of DKD before traditional indicators become abnormal.

Targeting endothelial function provides a new perspective for the treatment of DKD. In the final section, this review discusses targeting endothelial function for DKD treatment from three angles: direct anti-hyperglycemic therapy, non-antihyperglycemic interventions, and interventions targeting endothelial injury biomarkers. Drugs such as SGLT2 inhibitors and GLP-1 receptor agonists exert endothelial protective effects indirectly or directly through multiple mechanisms. However, due to limitations such as metabolic instability and multiple targets, current drug therapies targeting intercellular communication demonstrate limited clinical applicability. Based on the current evidence, we propose that endothelial dysfunction should be considered an early and central driver of DKD. Future research should focus on deciphering the heterogeneity of renal endothelial cells, developing diagnostic and therapeutic strategies based on extracellular vesicles, and elucidating key nodes in the multicellular communication network. Understanding DKD from the perspective of cellular dialogue will provide a critical theoretical foundation for early diagnosis and precise treatment.
